# Supplementary material for: Strain-level transmission inference across multi-kingdom metagenomic data using TRACS
Source: Nat Microbiol. 2026 Apr 24;11(6):1626–38. doi: 10.1038/s41564-026-02339-x (PMC13236588; doi:10.1038/s41564-026-02339-x)
Supplement: Supplementary file 2 — Reporting Summary [file 41564_2026_2339_MOESM2_ESM.pdf]

nature portfolio

Corresponding author(s): Gerry Tonkin-Hill

Last updated by author(s): Nov 19, 2025

# Reporting Summary

Nature Portfolio wishes to improve the reproducibility of the work that we publish. This form provides structure for consistency and transparency in reporting. For further information on Nature Portfolio policies, see our Editorial Policies and the Editorial Policy Checklist.

## Statistics

For all statistical analyses, confirm that the following items are present in the figure legend, table legend, main text, or Methods section.

| n/a | Confirmed | |
|---|---|---|
| ☐ | ☒ | The exact sample size (*n*) for each experimental group/condition, given as a discrete number and unit of measurement |
| ☐ | ☒ | A statement on whether measurements were taken from distinct samples or whether the same sample was measured repeatedly |
| ☐ | ☒ | The statistical test(s) used AND whether they are one- or two-sided<br>*Only common tests should be described solely by name; describe more complex techniques in the Methods section.* |
| ☐ | ☒ | A description of all covariates tested |
| ☐ | ☒ | A description of any assumptions or corrections, such as tests of normality and adjustment for multiple comparisons |
| ☐ | ☒ | A full description of the statistical parameters including central tendency (e.g. means) or other basic estimates (e.g. regression coefficient) AND variation (e.g. standard deviation) or associated estimates of uncertainty (e.g. confidence intervals) |
| ☐ | ☒ | For null hypothesis testing, the test statistic (e.g. *F*, *t*, *r*) with confidence intervals, effect sizes, degrees of freedom and *P* value noted<br>*Give P values as exact values whenever suitable.* |
| ☐ | ☒ | For Bayesian analysis, information on the choice of priors and Markov chain Monte Carlo settings |
| ☐ | ☒ | For hierarchical and complex designs, identification of the appropriate level for tests and full reporting of outcomes |
| ☐ | ☒ | Estimates of effect sizes (e.g. Cohen's *d*, Pearson's *r*), indicating how they were calculated |

*Our web collection on statistics for biologists contains articles on many of the points above.*

## Software and code

Policy information about availability of computer code

| | |
|---|---|
| Data collection | Fastq-dl (https://github.com/rpetit3/fastq-dl) v2.0 was used to download all sequencing reads from the ENA. |
| Data analysis | All code needed to reproduce the analyses, including the version numbers of third-party software, is available under an MIT open source license on GitHub at https://github.com/gtonkinhill/tracs/ and https://github.com/gtonkinhill/tracs_manuscript. |

For manuscripts utilizing custom algorithms or software that are central to the research but not yet described in published literature, software must be made available to editors and reviewers. We strongly encourage code deposition in a community repository (e.g. GitHub). See the Nature Portfolio guidelines for submitting code & software for further information.

## Data

Policy information about availability of data

All manuscripts must include a data availability statement. This statement should provide the following information, where applicable:

- Accession codes, unique identifiers, or web links for publicly available datasets
- A description of any restrictions on data availability
- For clinical datasets or third party data, please ensure that the statement adheres to our policy

The ENA IDs of the 37 SARS-CoV-2 samples found to contain multiple distinct lineages are listed in the supplementary GitHub repository. All SARS-CoV-2 sequencing reads from the original study are available in the ENA under accession number ERP126512. The sequencing reads for S. pneumoniae and P. falciparum are available from the ENA under project codes PRJEB22771 and PRJNA482776 respectively. The deep metagenomic sequencing reads of gut samples from mothers and their

children are available from the ENA under the accession number ERP115334. The FMT triad samples included sequencing data published in the ENA under project code PRJEB47909. The sample accession codes used in all analyses are provided in the supplementary files on GitHub.

## Research involving human participants, their data, or biological material

Policy information about studies with human participants or human data. See also policy information about sex, gender (identity/presentation), and sexual orientation and race, ethnicity and racism.

| | |
|---|---|
| Reporting on sex and gender | Sex and gender were not considered in this study. |
| Reporting on race, ethnicity, or other socially relevant groupings | Race, ethnicity and other similar groupings were not considered in this study. |
| Population characteristics | The population characteristics are described in the original publications from which data was obtained. These are provided in the data availability section of the manuscript and the relevant study accession numbers are provided above. |
| Recruitment | No participants were recruited for this study. |
| Ethics oversight | Ethics was obtained and described in previous studies cited in the data availability section. No participants were recruited for this study. |

Note that full information on the approval of the study protocol must also be provided in the manuscript.

# Field-specific reporting

Please select the one below that is the best fit for your research. If you are not sure, read the appropriate sections before making your selection.

☒ Life sciences ☐ Behavioural & social sciences ☐ Ecological, evolutionary & environmental sciences

For a reference copy of the document with all sections, see nature.com/documents/nr-reporting-summary-flat.pdf

# Life sciences study design

All studies must disclose on these points even when the disclosure is negative.

| | |
|---|---|
| Sample size | No sample size calculation was performed |
| Data exclusions | All accessions corresponding to the studies provided in the data section and provided in the supplementary GitHub were used in the analyses. |
| Replication | The accuracy of the computational method was confirmed through the analyses of multiple well characterized datasets and via simulation |
| Randomization | This is not relevant for this study as the focus is on the development of a new computational and statistical method. |
| Blinding | Blinding was not performed as the focus is on the development of a new computational and statistical method. |

# Reporting for specific materials, systems and methods

We require information from authors about some types of materials, experimental systems and methods used in many studies. Here, indicate whether each material, system or method listed is relevant to your study. If you are not sure if a list item applies to your research, read the appropriate section before selecting a response.

## Materials & experimental systems

| n/a | Involved in the study | |
|---|---|---|
| ☒ | ☐ | Antibodies |
| ☒ | ☐ | Eukaryotic cell lines |
| ☒ | ☐ | Palaeontology and archaeology |
| ☒ | ☐ | Animals and other organisms |
| ☒ | ☐ | Clinical data |
| ☒ | ☐ | Dual use research of concern |
| ☒ | ☐ | Plants |

## Methods

| n/a | Involved in the study | |
|---|---|---|
| ☒ | ☐ | ChIP-seq |
| ☒ | ☐ | Flow cytometry |
| ☒ | ☐ | MRI-based neuroimaging |

## Plants

| | |
|---|---|
| Seed stocks | *Report on the source of all seed stocks or other plant material used. If applicable, state the seed stock centre and catalogue number. If plant specimens were collected from the field, describe the collection location, date and sampling procedures.* |
| Novel plant genotypes | *Describe the methods by which all novel plant genotypes were produced. This includes those generated by transgenic approaches, gene editing, chemical/radiation-based mutagenesis and hybridization. For transgenic lines, describe the transformation method, the number of independent lines analyzed and the generation upon which experiments were performed. For gene-edited lines, describe the editor used, the endogenous sequence targeted for editing, the targeting guide RNA sequence (if applicable) and how the editor was applied.* |
| Authentication | *Describe any authentication procedures for each seed stock used or novel genotype generated. Describe any experiments used to assess the effect of a mutation and, where applicable, how potential secondary effects (e.g. second site T-DNA insertions, mosiacism, off-target gene editing) were examined.* |
